# EZSCAN for undiagnosed type 2 diabetes mellitus: A systematic review and meta-analysis

**DOI:** 10.1371/journal.pone.0187297

**Published:** 2017-10-30

**Authors:** Antonio Bernabe-Ortiz, Andrea Ruiz-Alejos, J. Jaime Miranda, Rohini Mathur, Pablo Perel, Liam Smeeth

**Affiliations:** 1 CRONICAS Center of Excellence in Chronic Diseases, Universidad Peruana Cayetano Heredia, Lima, Peru; 2 Faculty of Epidemiology and Population Health, London School of Hygiene and Tropical Medicine, London, United Kingdom; 3 Escuela de Medicina, Universidad Peruana de Ciencias Aplicadas–UPC, Lima, Perú; 4 Department of Medicine, School of Medicine, Universidad Peruana Cayetano Heredia, Lima, Peru; Florida International University Herbert Wertheim College of Medicine, UNITED STATES

## Abstract

**Objectives:**

The EZSCAN is a non-invasive device that, by evaluating sweat gland function, may detect subjects with type 2 diabetes mellitus (T2DM). The aim of the study was to conduct a systematic review and meta-analysis including studies assessing the performance of the EZSCAN for detecting cases of undiagnosed T2DM.

**Methodology/Principal findings:**

We searched for observational studies including diagnostic accuracy and performance results assessing EZSCAN for detecting cases of undiagnosed T2DM. OVID (Medline, Embase, Global Health), CINAHL and SCOPUS databases, plus secondary resources, were searched until March 29, 2017. The following keywords were utilized for the systematic searching: type 2 diabetes mellitus, hyperglycemia, EZSCAN, SUDOSCAN, and sudomotor function. Two investigators extracted the information for meta-analysis and assessed the quality of the data using the Revised Version of the Quality Assessment of Diagnostic Accuracy Studies (QUADAS-2) checklist. Pooled estimates were obtained by fitting the logistic-normal random-effects model without covariates but random intercepts and using the Freeman-Tukey Arcsine Transformation to stabilize variances. Heterogeneity was also assessed using the I^***2***^ measure. Four studies (n = 7,720) were included, three of them used oral glucose tolerance test as the gold standard. Using Hierarchical Summary Receiver Operating Characteristic model, summary sensitivity was 72.0% (95%CI: 60.0%– 83.0%), whereas specificity was 56.0% (95%CI: 38.0%– 74.0%). Studies were very heterogeneous (I^***2***^ for sensitivity: 79.2% and for specificity: 99.1%) regarding the inclusion criteria and bias was present mainly due to participants selection.

**Conclusions:**

The sensitivity of EZSCAN for detecting cases of undiagnosed T2DM seems to be acceptable, but evidence of high heterogeneity and participant selection bias was detected in most of the studies included. More studies are needed to evaluate the performance of the EZSCAN for undiagnosed T2DM screening, especially at the population level.

## Introduction

Worldwide, the burden of type 2 diabetes mellitus (T2DM) is rising rapidly. Currently, approximately 9% of adults in the world are living with T2DM [1, 2]. Many of the consequences of T2DM affect mainly low- and middle-income countries (LMIC): 1.5 million deaths worldwide were directly attributable to T2DM in 2012, and more than 80% of these deaths occurred in LMIC [3, 4]. In addition, about USD$ 548 billion in healthcare expenditures were due to T2DM in 2013 [5], imposing a large economic burden on individuals and families as well as health systems, particularly in resource-constrained settings.

Oral glucose tolerance test (OGTT) is considered the gold standard for T2DM diagnosis according to guidelines [6]. However, conventionally, fasting glucose (FG) is used in most of healthcare facilities. OGTT and FG require 8 hours of fasting and, in addition, OGTT also needs the participant to drink a 75-gram glucose solution and wait two hours before a second blood sample is obtained. In 2009, the American Diabetes Association suggested that glycated hemoglobin (HbA1c) could be used as a diagnostic tool for T2DM [7]. HbA1c does not require fasting, but can be expensive and requires a certified laboratory process [8]. Despite the recommended cutoff of 6.5% (48 mmol/mol) for T2DM diagnosis [9], discrepancies between HbA1c and glycemia in different racial and ethnic groups have been described [10–13].

An important approach to prevent or delay diabetes complications is to identify those individuals with undiagnosed T2DM [14]. Although universal T2DM screening at the population level is not practical; there are alternative methods reported in the literature. As early damage of small nerves can be found since the onset of T2DM [15], some devices have emerged to assess small-fiber autonomic dysfunction [16]. Among these devices, the EZSCAN (Impeto Medical, Paris, France), a non-invasive device that performs electrochemical reactions of eccrine sweat glands, may help to detect participants with diabetes mellitus [17, 18]. The advantage of the EZSCAN is that its use does not require trained personnel, delivers result quickly, and does not require active participation of the participants (i.e. fasting). Some studies have evaluated the potential impact of this device in pre-diabetes, dysglycemia and T2DM screening [17, 19, 20], but there is limited information regarding its potential for detecting cases of undiagnosed T2DM. Consequently, we conducted a systematic review and meta-analysis of observational studies to assess the performance of the EZSCAN for undiagnosed T2DM. Our hypothesis was focused on sensitivity, expecting at least a performance of 75%.

## Materials and methods

### Study selection

We searched for observational studies including diagnostic accuracy results assessing EZSCAN for undiagnosed T2DM, conducted in different parts of the world, but reported in English. Studies were excluded if they were only abstracts or review articles, enrolled individuals aged <18 years or cases with type 1 diabetes mellitus, and defined type 2 diabetes mellitus (T2DM) by using blood markers other than OGTT or FG (i.e. HbA1c). The rationale for this decision was based on discrepancies between HbA1c and glycemia in different racial and ethnic groups and that HbA1c is not commonly used for undiagnosed T2DM.

### Data sources and searches

A comprehensive literature search using the Ovid database (PubMed-Medline, Embase, Global Health, and Health Management Information Consortium) as well as CINAHL, and SCOPUS, until March 29, 2017, was conducted. The following keywords were utilized for the systematic searching: type 2 diabetes mellitus, hyperglycemia, EZSCAN, SUDOSCAN, and sudomotor function [16]. The term SUDOSCAN was also included in the search strategy as it uses the same principle (i.e. sudomotor function assessment) for detecting diabetic neuropathy [21, 22]. The search strategy of Ovid is available in S1 Table. The Impeto Medical website was also searched to find other published manuscripts [19].

### Data extraction and quality assessment

Titles and abstracts of retrieved articles were reviewed independently by two investigators to select potentially relevant articles, and disagreements were discussed and solved by consensus. Using a standardized data extraction form, we collected information on lead author, publication year, country, study design, inclusion criteria, used gold standard, sample size, mean age, percentage of male participants, and different indicators of the performance of the EZSCAN to detect undiagnosed T2DM (outcome, area under the curve, cut-off, sensitivity, specificity, among others).

Quality assessment of individual studies was performed to identify potential sources of bias and to limit, if possible, the effect of these biases on the conclusions of the review. For this, the Revised Version of the Quality Assessment of Diagnostic Accuracy Studies (QUADAS-2) checklist was used [23]. This tool included risk of bias assessment (i.e. participant selection, index test, reference standard, and flow and timing) as well as applicability.

### Data synthesis and analysis

The primary outcome of interest was undiagnosed T2DM (i.e. newly-diagnosed T2DM) identified by OGTT or FG. Secondary outcomes included other glucose metabolism disorders such as impaired glucose tolerance, impaired fasting glucose and, dysglycemia.

Statistical analyses were performed using Stata version 13 for Windows (StataCorp, College Station, TX, US). Our systematic review followed the Preferred Reporting Items for Systematic Review and Meta-Analysis (PRISMA, See S1 Checklist), the Guidelines for Meta-Analyses and Systematic Reviews of Observational Studies (MOOSE) [24] as well as the Cochrane Handbook for Diagnostic Test Accuracy Reviews [25]. Initially, the studies included in the systematic review were described, including: publication year, country, study design, inclusion criteria, gold standard, sample size, mean age, and proportion of males. In addition, the performance of the EZSCAN in each study was tabulated, and the area under the receiver operating characteristic (ROC) curve (AUC), best cut-off, sensitivity, and specificity, and their respective 95% confidence intervals (95%CI) were reported, if available.

A meta-analysis of the performance of the EZSCAN was conducted using data from studies with undiagnosed T2DM as outcome. Information used in meta-analysis was taken as proposed by manuscripts according to the best EZSCAN threshold cut-off reported. The “*metaprop”* command in STATA was used to estimate sensitivity, specificity and positive (PPV) and negative (NPV) predictive values and their respective 95%CI [26]. The “*metaprop*” command obtains a pooled estimate as a weighted average, by fitting the logistic-normal random-effects model without covariates but random intercepts. The pooled estimate was then calculated using the Freeman-Tukey Arcsine Transformation to stabilize the variances as suggested in literature [27]. In addition, a graph containing the plot of the Hierarchical Summary Receiver Operating Characteristic (HS-ROC) model [28], a summary point of sensitivity and specificity and the 95% confidence region for that point was obtained by using the “*metandiplot*” command [29]. Heterogeneity of estimates and 95%CI was determined using the I^2^ measure [30].

## Results

### Study characteristics

**Fig 1:**
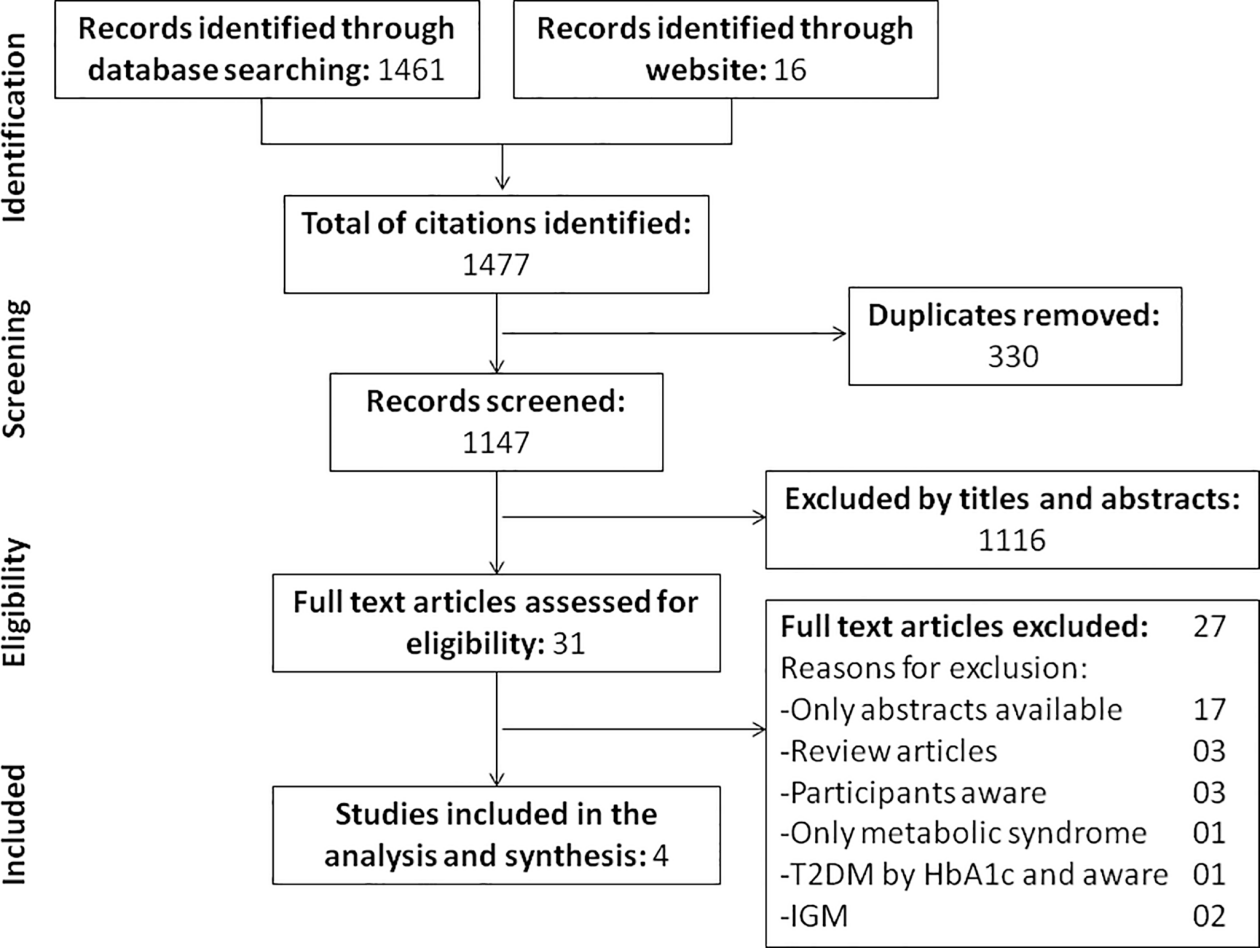
Flowchart of database searches and articles included in the systematic review. T2DM: Type 2 diabetes mellitus, HbA1c = glycated hemoglobin, IGM = impaired glucose metabolism.

A total of 1,461 citations were identified through our systematic search, with a further 16 citations identified using the Impeto Medical website. After excluding duplicates (n = 330), a total of 1,147 citations were independently screened, of which 31 were retrieved for detailed assessment (agreement between reviewers, 97.2%, kappa = 0.61, p<0.001). Of the 31 revised manuscripts, 27 did not fit our inclusion criteria (Fig 1); therefore, four studies were included in the systematic review.

The characteristics of the studies included in the systematic review are shown in Table 1. All the four studies were cross-sectional in nature. A total of 7,720 individuals were included from all the studies, but 5,824 subjects came from a single study [31]. This latter study enrolled individuals from the general population, whereas the remaining three studies recruited participants at clinics, mainly individuals going for healthy check-ups.

**Table 1:** Characteristics of the studies included in the systematic review.

Study, publication year	Country	Study design	Inclusion criteria	Gold standard	Size	Mean age	% male
Chen X, 2015 [32]	China	Cross-sectional	Subjects in routine health check visiting a Community Hospital, at risk of T2DM (age ≥ 45 years).	OGTT	270	58.6	32%
Ramachadran A, 2010 [33]	India	Cross-sectional	Individuals in specific clinics aged between 21–75 years.	OGTT	212	43.4	45%
Sanchez-Hernandez O, 2015 [34]	Mexico	Cross-sectional	Individuals recruited in a clinic in Mexico; ≥18 years, apparently healthy and attending a full check-up.	FG	1,414	44.7	50%
Yang Z, 2013 [31]	China	Cross-sectional	Individuals from two communities in Shanghai aged 40+ years.	OGTT	5,824	58.3	40%

FG = fasting glucose; OGTT = oral glucose tolerance test.

### Risk of bias

Overall, participant selection bias was present in 3 out of 4 of the studies included in the meta-analysis [32–34]: individuals under healthy check-ups were enrolled in the original studies (S2 Table). In addition, flow and timing was unclear in the same three studies, and the gold standard (i.e. OGTT) was not used in one of the studies [34].

### Meta-analysis: EZSCAN performance for undiagnosed T2DM

Undiagnosed T2DM was the outcome of interest in the four studies (Table 2). Other outcomes evaluated in these papers included impaired glucose tolerance [32, 33], impaired fasting glucose [34] and dysglycemia [31].

**Table 2:** Performance of the EZScan in the studies included in the systematic review.

Study, publication year	Outcome	AUC	Cut-off	Sensitivity	Specificity
Chen X, 2015 [32]	IGT	78% (72%–83%)	37%	82% (72%–90%)	63% (55%–71%)
Chen X, 2015 [32]	T2DM	53% (43%–62%)	50%	53% (36%–69%)	59% (47%–70%)
Ramachadran A, 2010 [33]	IGT	—	50%	70% (not reported)	54% (not reported)
Ramachadran A, 2010 [33]	T2DM	—	50%	75% (not reported)	54% (not reported)
Sanchez-Hernandez O, 2015 [34]	IFG	65% (not reported)	27%	69% (not reported)	56% (not reported)
Sanchez-Hernandez O, 2015 [34]	T2DM	73% (not reported)	34%	73% (not reported)	70% (not reported)
Yang Z, 2013 [31]	IFG, IGT or T2DM	—	30%	73% (71%-75%)	46% (45%-48%)
Yang Z, 2013 [31]	T2DM	—	30%	81% (78%-83%)	43% (42%-44%)

IFG = Impaired fasting glucose; IGT = Impaired glucose tolerance; T2DM = type 2 diabetes mellitus; AUC = area under the curve.

Values in brackets are 95% confidence intervals (95%CI).

When undiagnosed T2DM was the outcome, only two studies reported results of AUC ranging from 53% to 73% [32, 34]. In addition, 2 studies used 50% as the suggested EZSCAN cut-off for undiagnosed T2DM screening [32, 33], whereas one used 34% [34], and the last one utilized 30% [31]. Sensitivity varied from 53% to 81%, whilst specificity ranged from 43% to 70%. Finally, positive predictive values (PPV) varied from 10% to 40%, whereas negative predictive values (NPV) ranged from 71% to 98%.

**Fig 2:**
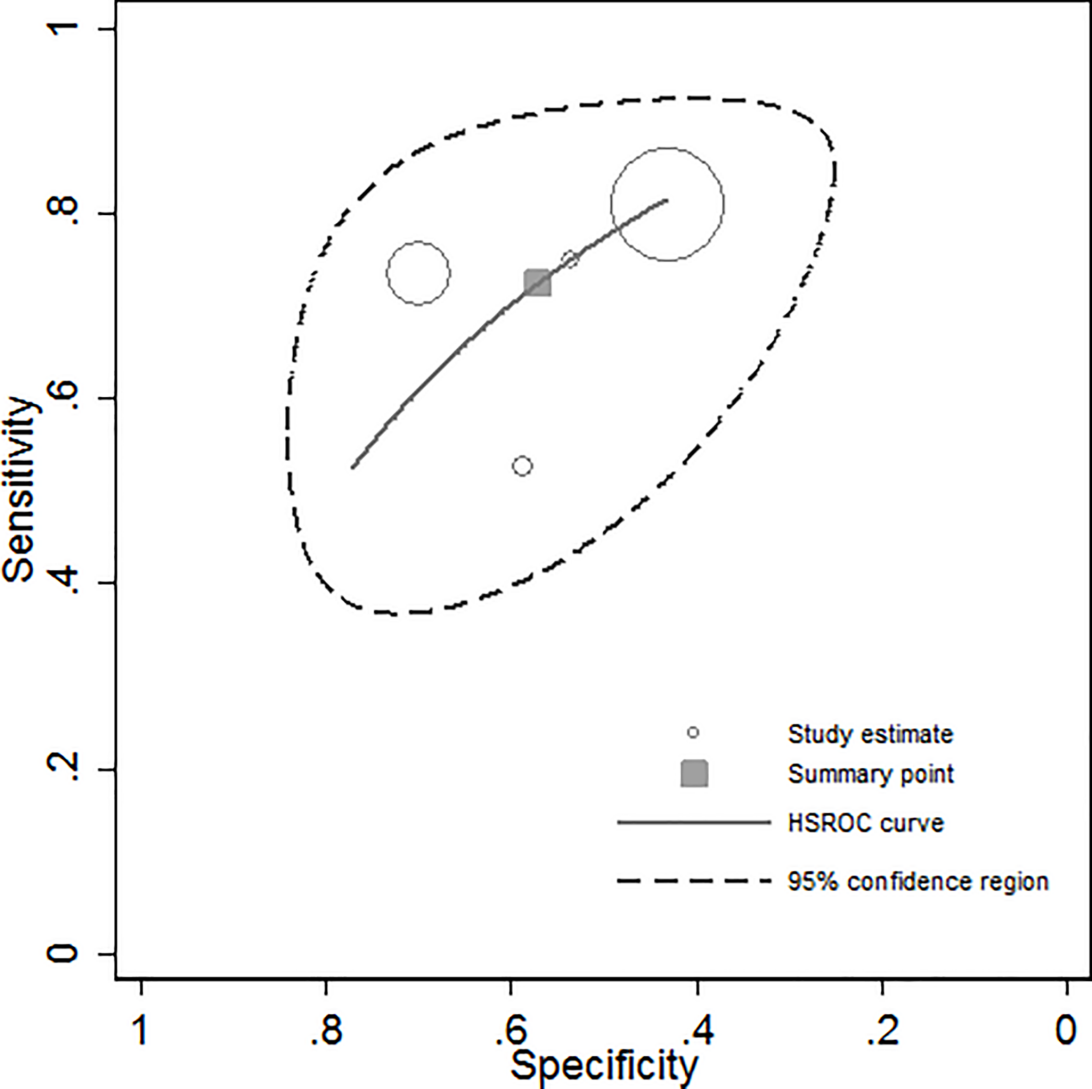
Performance of EZScan in the screening of T2DM: Meta-analysis using HSROC. Sensitivity = 72.0% (95%CI: 60.0%–83.0%); specificity = 56.0% (95%CI: 38.0%–74.0%); likelihood ratio positive = 1.68 (95%CI: 1.35–2.10); likelihood ratio negative = 0.48 (95%CI: 0.36–0.66); DOR = 3.49 (95%CI: 2.18–5.57). HSROC curve is shown only for sensitivities and specificities at least as large as the smallest study-specific estimates.

When using HS-ROC (Fig 2), summary sensitivity was 72.0% (95%CI: 60.0%– 83.0%), specificity was 56.0% (95%CI: 38.0%– 74.0%), PPV was 24% (95%CI: 12.0%– 37.0%), and NPV was 89% (95%CI: 82.0%– 97.0%). In addition, positive and negative likelihood ratios were 1.68 (95%CI: 1.35–2.10) and 0.48 (95%CI: 0.36–0.66), respectively, whereas the DOR was 3.49 (95%CI: 2.18–5.57). Heterogeneity for sensitivity was 79.2% (95%CI: 44.0%– 92.0%), whereas for specificity was 99.1% (95%CI: 98.5%– 99.6%).

## Discussion

### Summary of evidence

According to the results of this systematic review and meta-analysis, the performance of the EZSCAN in the detection of cases undiagnosed T2DM can be considered acceptable especially in the case of sensitivity, and even comparable to different well-known T2DM risk scores [35, 36]. To put in context our findings, the sensitivity of HbA1c, using a cut-off ≥6.5% (48 mmol/mol), for detecting undiagnosed diabetes was 52.8% using OGTT as the gold standard [37]. Thus, apparently, the EZSCAN might perform better that HbA1c although other studies are needed to corroborate this.

There are, however, some limitations that need to be highlighted. First, there is a risk of bias based on participant selection that can complicate extrapolation of results: many of the studies were performed in clinical context (i.e. clinical check-ups) instead of using population level assessments. Second, a high level of heterogeneity between studies was found (greater than 75%) in all estimations (i.e. sensitivity, specificity, etc). Since a small number of studies were included in the meta-analysis; results need to be cautiously interpreted despite of the fact that random effect models were used in calculations [38]. In addition, heterogeneity in results of the EZSCAN performance can be secondary to characteristics of the context and individuals: predictive values as well as likelihood ratios can depend on baseline risk of evaluated subjects. For example, the association of body mass index–one of the variables used in scoring individuals through EZSCAN–with the risk of diabetes may vary in different populations [39], and explain variability found in this report. Finally, although there is a suggested EZSCAN cut-off for defining T2DM in the population (50%), our results showed heterogeneity of this cut-off between studies and populations assessed: only two studies used the proposed 50% cut-off [32, 33], whereas the other studies were below that value. Thus, the device needs to be validated in different populations.

The principle of the EZSCAN, based on the evaluation of sudomotor function, relies on the assessment of chloride concentrations using reverse iontophoresis and chronoamperometry to detect insulin resistance and T2DM [18]. The EZSCAN has showed reproducible results in several conditions with low impact of usual physiological variations due to its focus on chloride concentrations, instead of sweat rates as used by other methods [40]. This device deliver results rapidly (i.e. in 2 to 3 minutes) and does not require invasive blood testing or fasting. Moreover, no safety problems have been reported during its use. Of note, although the EZSCAN has been designed to detect individuals with undiagnosed T2DM [18], some of the studies have focused on the ability of the device to detect impaired fasting glucose [17, 41, 42], dysglycemia [31, 33], metabolic syndrome [20], or even, complications related to T2DM [43, 44]. On the other hand, a relatively recent paper combined the performance of this device with conventional risk scores and reported limited improvement in the model given by the sum of EZSCAN plus risk score in Chinese population [31]. However, authors claimed that other studies are needed to determine the clinical relevance of EZSCAN in detecting cases of diabetes.

### Public health relevance

Sensitivity and specificity estimates from this review may be used to better understand EZSCAN testing in real practice. For example, in a given setting with a prevalence of undiagnosed T2DM of 10% and assuming a cut-off value of 50% as suggested by the provider, if 1,000 individuals were screened using the EZSCAN, based on tool sensitivity, the device would detect 72 undiagnosed T2DM cases and 28 would be missing (false negatives). On the other hand, from the 900 individuals without the disease, 396 would be false positives and classified as having T2DM with the subsequent need of a confirmatory test. Thus, we would only need to perform 468 OGTT for those positive for EZSCAN, instead of the total population. If the prevalence were higher (i.e. 20% instead of 10%), of the 1,000 individuals, the device would detect 144 individuals based on its sensitivity, but 56 cases would be missing (false negatives). Of the 800 subjects without the disease, 352 would be false positives and classified as having T2DM with the need of a confirmatory test. Therefore, 496 OGTT would be needed but missing 56 cases as false negatives. On the other hand, summary estimates of the positive and negative likelihood ratios were very similar to values compatible with minimal change in the likelihood of disease. Thus, if positive and negative likelihood ratios of >10 and <0.1, respectively, were available, this would provide strong evidence to confirm and discard undiagnosed T2DM [45].

Using EZSCAN for detecting undiagnosed T2DM cases can have some advantages including the short time spent in conducting the test, the fact that fasting is not required, and the repeated used of the device can compensate its cost. However, some disadvantages also arise. Although, the EZSCAN can potentially reduce the resources implied in assessing populations for detecting T2DM cases, the number of false negatives (i.e. individuals with undiagnosed T2DM that are not detected by the device) increased when the prevalence of diabetes increased. On the other hand, literature suggested that EZSCAN cutoff should be estimated by each population instead of only using the cut-off given by the provider [31, 34, 46].

To our knowledge there is no information regarding the cost-effectiveness of the EZSCAN for detecting one undiagnosed case of T2DM in addition to the lack of data related to the potential performance for future risk of T2DM. Only one study has assessed the utility of this device longitudinally (2-year follow-up) but in a small sample [17]. In this study, the authors found an association between the EZSCAN score and T2DM progression although results needed further confirmation. Thus, the EZSCAN might have potential implications for T2DM prevention although population-based validation may be necessary to define appropriate cut-off for appropriate results interpretation.

### Limitations

One of the limitations of this review is the representativeness of the results characterized by bias in participants’ selection as well as the lack of a true gold standard in some of the studies (i.e. FG was used in one study instead of OGTT). In addition, characteristics of the study population were poorly reported and this is reflected in the quality assessment. As all the studies assessing EZSCAN were recently published (from 2010 and onwards); authors should have been utilized the Standards for Reporting Diagnostic Accuracy Studies (STARD) to guide their manuscripts’ writing [47]. Future studies should follow these guidelines to guarantee an appropriate reporting of diagnostic studies.

Given the limited number of studies assessed, EZSCAN threshold was not meta-analyzed as the performance of the diagnostic test depends on the population in which the test is used. Thus, for our analyses, pooled sensitivity and specificity were calculated using the best cut-off reported by studies and not the same in all cases. In addition, there is limited data evaluating the potential impact of EZSCAN for undiagnosed T2DM at the population level. Future studies should be focused on population-based samples instead of referral health facilities, but also in different ethnic groups as only studies from China and India were used in this review. A study from Mexican population was also included in the meta-analysis, but the sample was biased and FG was used as gold standard [34]. Moreover, as the number of studies included in the analysis was small, publication bias was not assessed (usual tests for publication bias are underpowered when <10 studies are evaluated).

In summary, the sensitivity of the EZSCAN for undiagnosed T2DM screening seems to be acceptable but the evidence is limited because of the presence of participant selection bias in most of the included studies in the meta-analysis. The performance of the EZSCAN warrants confirmation in different populations, using the appropriate gold standard, and population-based samples. Moreover, adequate report of findings and longitudinal utility of the EZSCAN is also compulsory.

## Supporting information

S1 Checklist PRISMA Checklist information. (DOC)

S1 Table Search strategy and databases included for EZScan used in OVID. (DOC)

S2 Table Quality assessment of the studies included in the systematic review (QUADAS-2). (DOC)
