# Supplementary material for: EZSCAN for undiagnosed type 2 diabetes mellitus: A systematic review and meta-analysis
Source: PLoS One. 2017 Oct 30;12(10):e0187297. doi: 10.1371/journal.pone.0187297 (PMC5662214; doi:10.1371/journal.pone.0187297)
Supplement: S1 Table — (DOC) [file pone.0187297.s002.doc]

**S1 Table: Search strategy and databases included for EZScan used in OVID**

**Databases included:**

Global Health 1910 to 2017 Week 11; HMIC Health Management Information Consortium 1979 to January 2017; Journals@Ovid Full Text March 29, 2017; Ovid MEDLINE(R) 1946 to March Week 4 2017; PsycINFO 1806 to March Week 3 2017; Embase 1974 to 2016 March 29.

| **#** | **Searches** | **Results** |
| --- | --- | --- |
| 1 | type 2 diabetes.mp. | 382,642 |
| 2 | diabet*.mp. | 2,257,087 |
| 3 | hyperglycem*.mp. | 225,801 |
| 4 | T2D*.mp. | 69,504 |
| 5 | DBM.mp | 4,657 |
| 6 | (#1 or #2 or #3 or #4 or #5) | 2,329,381 |
| 7 | exp Diabetes Mellitus | 1,249,251 |
| 8 | exp Diabetes Mellitus, Type 2/ | 311,830 |
| 9 | (#7 or #8) | 1,249,251 |
| 10 | (#6 or #9) | 2,335,634 |
| 11 | EZScan.mp. | 81 |
| 12 | SUDOSCAN.mp | 167 |
| 13 | sudom*.mp | 4,932 |
| 14 | (#11 or #12 or #13) | 5,021 |
| **15** | **(#10 and #14)** | **1,345** |
