# Supplementary material for: EZSCAN for undiagnosed type 2 diabetes mellitus: A systematic review and meta-analysis
Source: PLoS One. 2017 Oct 30;12(10):e0187297. doi: 10.1371/journal.pone.0187297 (PMC5662214; doi:10.1371/journal.pone.0187297)
Supplement: S2 Table — (DOC) [file pone.0187297.s003.doc]

**S2 Table: Quality assessment of the studies included in the systematic review (QUADAS-2)**

| **Study, publication year** | **Risk of bias** | | | | **Applicability** | | |
| --- | --- | --- | --- | --- | --- | --- | --- |
|  | **Patient selection** | **Index**  **test** | **Reference standard** | **Flow and timing** | **Patient selection** | **Index**  **test** | **Reference standard** |
| Chen X, 2015 | High | Low | Low | Unclear | Unclear | Low | Low |
| Ramachadran A, 2010 | High | Low | Low | Unclear | High | Low | Low |
| Sanchez-Hernandez O, 2015 | High | Unclear | High | Unclear | High | Unclear | High |
| Yang Z, 2013 | Low | Low | Low | Low | Low | Low | Low |
